# Melanoma Management during the COVID-19 Pandemic Emergency: A Literature Review and Single-Center Experience

**DOI:** 10.3390/cancers13236071

**Published:** 2021-12-02

**Authors:** Caterina Cariti, Martina Merli, Gianluca Avallone, Marco Rubatto, Elena Marra, Paolo Fava, Virginia Caliendo, Franco Picciotto, Giulio Gualdi, Ignazio Stanganelli, Maria Teresa Fierro, Simone Ribero, Pietro Quaglino

**Affiliations:** 1 Department of Medical Sciences, Dermatology Clinic, University of Turin, 10126 Turin, Italy; caterina.cariti@gmail.com (C.C.); gianluca.avallone@hotmail.it (G.A.); marco.rubatto@edu.unito.it (M.R.); emarra@cittadellasalute.it (E.M.); fava_paolo@yahoo.it (P.F.); mariateresa.fierro@unito.it (M.T.F.); simone.ribero@unito.it (S.R.); pietro.quaglino@unito.it (P.Q.); 2 Dermatologic Surgery Department, Surgery Department, University Hospital, 10126 Turin, Italy; virginia.caliendo@unito.it (V.C.); fpicciotto@cittadellasalute.to.it (F.P.); 3 Department of Medicine and Ageing Science, Dermatologic Clinic, “G. D’Annunzio” University, 66100 Chieti, Italy; giuliogualdi@libero.it; 4 Skin Cancer Unit, IRCCS-IRST Scientific Institute of Romagna for the Study and Treatment of Cancer, Meldola and University of Parma, 43121 Parma, Italy; ignazio.stanganelli@unipr.it

**Keywords:** melanoma, COVID-19, management, pandemic, teledermatology, diagnostic delay, immunotherapy, vaccination, lymph adenopathy

## Abstract

**Simple Summary:**

COVID-19 is a highly contagious infection caused by severe acute respiratory syndrome coronavirus 2 (SARS-CoV-2). In March 2020, the World Health Organization (WHO) declared that COVID-19 had become a pandemic; since then, several elective clinical and surgical activities have been postponed to reduce the risk of nosocomial infection. This has influenced the diagnosis and management of many diseases, including melanoma. The aim of our literature review was to evaluate whether the management of melanoma has been changed by the outbreak of COVID-19, and if so, what the consequences of these changes are. The main topics in this literature review are the screening of suspicious lesions, diagnosis of primary melanoma, and the management of early-stage and advanced melanomas in the COVID-19 era. We also reported the experience of our dermatological clinic in Turin, one of the most affected areas in Italy.

**Abstract:**

Background: The current COVID-19 pandemic has influenced the modus operandi of all fields of medicine, significantly impacting patients with oncological diseases and multiple comorbidities. Thus, in recent months, the establishment of melanoma management during the emergency has become a major area of interest. In addition to original articles, case reports and specific guidelines for the period have been developed. Purpose: This article aims to evaluate whether melanoma management has been changed by the outbreak of COVID-19, and if so, what the consequences are. We summarized the main issues concerning the screening of suspicious lesions, the diagnosis of primary melanoma, and the management of early-stage and advanced melanomas during the pandemic. Additionally, we report on the experience of our dermatological clinic in northern Italy. Methods: We performed a literature review evaluating articles on melanomas and COVID-19 published in the last two years on PubMed, as well as considering publications by major healthcare organizations. Concerning oncological practice in our center, we collected data on surgical and therapeutic procedures in patients with a melanoma performed during the first months of the pandemic. Conclusions: During the emergency period, the evaluation of suspicious skin lesions was ensured as much as possible. However, the reduced level of access to medical care led to a documented delay in the diagnosis of new melanomas. When detected, the management of early-stage and advanced melanomas was fully guaranteed, whereas the follow-up visits of disease-free patients have been postponed or replaced with a teleconsultation when possible.

## 1. Introduction

The severe acute respiratory syndrome coronavirus 2 (SARS-CoV-2) pandemic has increased the workload of the health system in the last two years, which has also presented a global challenge for dermatologists. Several elective clinical and surgical activities have been postponed to reduce the risk of nosocomial infections in patients and healthcare workers. This does not concern oncological patients, whose medical care has been guaranteed. However, cancer screening programs have clearly been interrupted by the onset of COVID-19 [1].

An early diagnosis is essential for the survival of patients with a melanoma [2]. The position statement issued by the European Academy of Dermatology and Venereology (EADV) affirmed that an in-person physical examination is obligatory for an accurate diagnosis of suspicious new cutaneous lesions, and that dermoscopy remains the gold standard for melanoma diagnosis. Although there have been no reports of COVID-19 transmission via dermatoscopes, the device must be carefully disinfected between patients and adequate personal protective equipment must be worn by physicians and patients [3]. In this context, the reduced level of access to medical care is a problem for dermatologists, due to the potential decrease in diagnoses of thin melanomas and delays in the presentation of patients with thick tumors. A survey conducted by the International Dermoscopy Society (IDS) showed that its members had experienced a 75% reduction in daily work activity since the beginning of pandemic, and remarkably, the number of melanomas diagnosed in these months was practically zero for more than half of them (56.78%) [4]. In addition to the devastating consequences for patients, delays in the treatment of melanomas can have a profound impact on the economic burden of this disease, because advanced melanomas have higher healthcare costs than earlier-stage melanomas [5].

**Figure 1:**
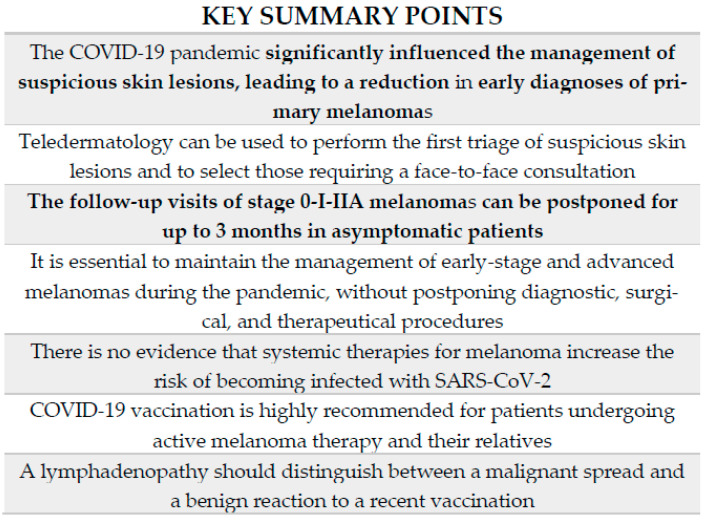
The main topics discussed in the literature review.

In this paper, we examine the main topics concerning the management of melanomas during this healthcare emergency, focusing on the importance of teledermatology, the diagnostic delay of melanomas, and how daily oncology practice has been impacted by the COVID-19 pandemic (Figure 1). We also report on the experience of our clinical center, which is situated in one of the most affected areas in Italy.

## 2. The Increased Importance of Teledermatology Caused by COVID-19

The role of telemedicine has been discussed at length as a valid medical approach during the COVID-19 era. Telemedicine can be defined as the application of diagnostic and therapeutic tools at a distance from the patient as an alternative to a face-to-face consultation; it can be used in several fields of medicine.

Teledermatology can be classified into real-time teledermatology (VTC—live video consultation) and store-and-forward teledermatology (SAF—image transmission by the patient). Both types of teledermatology have facilitated remote dermatological assistance during the pandemic for chronic diseases and the appearance/modification of skin lesions [6]. The IDS questioned 678 of its members, from 52 different countries, about their activity during the emergency period. In total, 27.73% of respondents affirmed that telemedicine represented an important method of performing consultations; the number of unofficial teleconsultations (e.g., by mail, SMS, Skype^®^, WhatsApp^®^, Messenger^®^, etc.) they were asked to conduct increased by 83.33% during the COVID-19 pandemic [4]. A small-scale, randomized controlled trial comparing all teledermatology modalities and face-to-face consultations found that the diagnosis and recommended course of treatment was the same in 85% and 78% of cases, respectively [7]. Even if teledermatology can correctly identify the majority of malignant lesions, the diagnostic accuracy of face-to-face consultations remains higher than that of remote consultations [8].

Smartphone-based teledermoscopy, an extension of teledermatology, has improved in recent years due to the development of mobile applications and devices. Veronese et al. tested an inexpensive and easy-to-use smartphone microscope device; overall, they obtained an accuracy of 83.9% for the diagnoses of skin lesions based on images acquired through the device [6]. Considering these useful tools, teledermoscopy in association with teledermatology can be used to perform the first triage of skin lesions, helping the clinician to select patients who require a face-to-face consultation [8].

Nahm et al. described an example of telemedicine’s usefulness during the COVID-19-enforced lockdown. They reported the case of a 66-year-old man with two biopsy-proven melanomas that were diagnosed in situ before the pandemic. The patient refused the exeresis of the two lesions due to his fear of coronavirus infection. Hence, the patient was treated with a combination of topical imiquimod 5% cream, 5-fluorouracil 2% solution, and tretinoin 0.1% cream every day for a month. The follow-up consultations were conducted exclusively via video consultation, and the histological evaluation completed 3 months after the treatment showed a complete resolution of the lesions [9].

## 3. The Diagnostic Delay of Melanomas during the Emergency Period

The great concern regarding the diagnostic delay of melanomas, and generally tumors, has been reflected by the large number of studies conducted on this topic during the emergency period.

The number of first diagnoses of malignancy recorded during weeks 11 to 20 of 2020 was compared with the same periods from 2018 and 2019. The data were collected from seven secondary care hospital networks of northern and central Italy. In 2020, there were 2751 new diagnoses, representing a decrease of 44.9% compared to 2018 and 2019 (4991.5 cases on average). The weekly number of diagnoses, considering the beginning of lockdown as the baseline time, constantly decreased during the first 2 weeks. The main reduction in new diagnoses occurred at week 16 (64.6% decrease compared with the same week in 2018–2019) and, during the last 2 weeks, a new increase was observed. Of the total number of all missing cancer diagnoses, melanoma and nonmelanoma skin cancers represented 56.7% [10].

Longo and Peris reported a significant reduction in the diagnoses of primary melanomas between 1 January 2020 and 9 May 2020 in Reggio Emilia and Rome, respectively. The number of new primary melanomas detected during this timeframe in 2019 was 141 and 115 in Rome and Reggio Emilia, respectively, whereas in 2020, there were 62 and 28 new primary melanomas detected [11].

Similarly, a third-level center in northern Italy observed a significant 60% reduction in new melanoma diagnoses between 22 February 2020 and 3 May 2020 compared to the same timeframes in 2018 and 2019. The most frequent histotype analyzed during the lockdown period was in situ melanoma (66.7%), whereas superficial spreading melanoma was the most frequent during 2019 and 2018 (52.4%). This may reflect a loss of thick melanomas in 2020, that conversely had been removed in 2019 and 2018 [12].

Generally, in Italy from February 2020 to April 2020, the Intergruppo Melanoma Italiano (IMI) detected a substantial reduction in the number of first visits (−31.3%) and biopsies (−36.5%), with a decrease of approximately 25% in histological diagnoses, and a 22.9% reduction in wide local excisions in comparison to 2019. As a result, there was a 20.8% reduction in the number of patients starting systemic therapy [13].

The Italian data described above are in agreement with studies in other countries. Lallas et al. compared the observed and expected numbers of new melanomas, basal cell carcinomas (BCCs), and cutaneous squamous cell carcinomas (cSCCs) in 2020 in northern Greece. The expected incidence of each tumor was calculated as the mean of the previous 4 years (2016–2019), considering this figure to be stable in 2020. The total number of these new skin cancers was 30.1% lower than expected, with a 36.4% reduction in the number of expected melanoma diagnoses. The patients with a melanoma were significantly younger than those in previous years, reflecting the greater fear of COVID-19 among elderly individuals. Additionally, a significantly higher proportion of melanomas than expected were diagnosed at stages IIC, III, and IV [14]. 

A multicenter observational study performed in Spain analyzed all patients who underwent melanoma or cSCC surgery in the period between March and June 2020. The comparison with the same period in 2019 revealed that there was a 41% reduction in treated melanoma during the first lockdown, with fewer melanomas in situ (34.9% vs. 29%) and a slight increase in thick melanomas (>4 mm) (10.2% vs. 18.4%). Similar results were obtained for cSCC. Patient-related factors, such as age, living in a nursing home, and fear of infection with SARS-CoV-2, were associated with a greater Breslow thickness [15].

Conversely, a dermatology department in London reported that a high proportion of early-stage melanomas were diagnosed during the UK’s COVID-19 lockdown. There are several factors implicated in this higher melanoma detection-to-referral ratio, including the impact that the setting of anxiety and restricted healthcare services has on patient self-selection [16].

## 4. Melanoma Surgery and Disease-Free Patient Follow-Up during the Pandemic

According to the European Society for Medical Oncology (ESMO) [17] and National Comprehensive Cancer Network (NCCN) guidelines [18], the position statement of the EADV Melanoma Task Force affirmed that the excision of a suspicious lesion should be performed as soon as possible to remove everything that is clinically visible [3]. In addition, the NCCN suggests that performing a broad-shave biopsy for larger suspected melanomas in situ and lentigo maligna, preventing the need to perform a subsequent radicalization. Wide excision should be postponed for up to 3 months for melanomas in situ and invasive melanomas for which a previous biopsy has showed clear histopathological margins or a peripheral transection of the in situ component [18]. It has been noted that delaying the surgical excision of melanoma by one month or longer increases the proportion of large or thick tumors, resulting in a lower chance of overall survival. In this regard, Tejera-Vaquerizo and Nagore built a model based on melanoma rate of growth (ROG), i.e., the rate of increase in Breslow thickness (millimeters per month) from the time a subject first observes a suspicious lesion to its excision. This predictive model was used to understand how diagnostic delays may impact the prognosis of melanomas [19].

When possible, wide excision and sentinel lymph node biopsy (SLNB—for melanoma thicker than 0.8 mm) should be performed at the same time. Otherwise, SLNB may be delayed by up to 3 months [18], as it has been demonstrated that such an interval of time does not have a negative effect on disease-free and overall survival [20]. Regarding therapeutic lymph node dissection, the EADV’s position statement indicated that it should be limited to patients with clinically evident regional lymph node metastases [3]. Conversely, NCCN guidelines affirm that lymphadenectomy should also be delayed in cases of clinically evaluable lymph adenopathy when a systemic neoadjuvant treatment is possible. In such a case, surgery should be performed 8–9 weeks after starting neoadjuvant treatment. This is not applicable when the affected lymph node is adjacent to vital organs and/or there is a contraindication to systemic therapy or a previous neoadjuvant treatment failure [18]. Finally, the guidelines agree that high surgical priority should be given to all invasive primary melanomas, resectable stage III melanomas, and oligo-metastatic disease [3]. 

Regarding disease-free patients, and according to the EADV’s position statement, the clinical and radiological follow-up in stage 0-I-IIA melanoma can be postponed for up to 3 months in asymptomatic patients [3]. Moreover, no clear evidence of an increase in survival in stages IB and IIA has been reported in ultrasound-based follow-ups [21]. High-risk patients should continue to have physical and imaging examinations, particularly during the first 3 years after surgery on the primary tumor [3]. On the other hand, NCCN guidelines suggest a possible deferral of up to 6 months even for stages IIB/IIC. During this delayed time, self-examination once a month is highly recommended [18].

## 5. Advanced Melanoma Management and Immunotherapy in the COVID-19 Era

For adjuvant therapy and treatment of unresectable stage III or IV melanomas, the indication of EADV is the same in a non-emergency setting; the treatment of melanomas with approved drugs must be started within 12 weeks of surgery. Considering these patients at higher risk for a severe course of COVID-19 infection, antibodies against Programmed Cell Death Protein 1 (PD1) should be administered using the longest approved treatment schedule to decrease hospital admissions: pembrolizumab 400 mg every 6 weeks and nivolumab 480 mg every 4 weeks [3]. Given its favorable safety profile, a monotherapy with anti-PD1 should be preferred in the majority of patients requiring immunotherapy [22]. The addition of ipilimumab, an antibody targeting cytotoxic T-lymphocyte-associated protein 4 (CTLA-4), significantly increases the risk of immune-related adverse events (iRAEs) from 15%–20% to 50%–60% compared to anti-PD1 monotherapy; this often leads to the need for immunosuppressive therapies and hospitalization, which in turn potentially increases the rate of COVID-19 transmission and severe disease [23]. Among the complications caused by immune checkpoint inhibitors (ICIs), immunotherapy-related pneumonitis and COVID-19 pneumonia have overlapping clinical and radiological features, leading to significant challenges in differential diagnosis [24]. Accordingly, the combination of nivolumab and ipilimumab is recommended only in subsets of patients with specific clinical features, including symptomatic and asymptomatic cerebral metastases, elevated LDH levels, bulky disease, PD-L1 negativity, and mucosal and acral melanoma [3,25]. The CheckMate 511 regimen—that is, ipilimumab 1 mg/kg plus nivolumab 3 mg/kg—is the preferred course of treatment due to a significantly lower incidence of treatment-related grade 3–5 AEs [26].

In patients undergoing BRAF and MEK inhibitor treatments, hyperpyrexia must be considered primarily as an adverse event rather than an indicator of COVID-19 infection. For this reason, encorafenib combined with binimetinib is preferred when available compared to other regimens due to its lower fever incidence [27]. In cases of fever associated with grade 2 or higher dyspnea, diarrhea, or neurological symptoms that do not resolve after therapy discontinuation, patients should be tested for a SARS-CoV-2 infection [3].

Concerning intracranial metastases, in cases of neurological deterioration or surgery, such as palliative care following treatment delays, stereotactic radiosurgery should be considered [25]. 

According to NCCN guidelines, in patients progressing beyond standard immune checkpoint blockade and targeted therapy, hospice care should be considered, because chemotherapy only provides a limited benefit. Oral temozolomide is the preferred option for palliative care cases [28].

As indicated by the EADV’s recommendations, all patients undergoing surgery, radiotherapy, chemotherapy, or immunotherapy must be tested for COVID-19 infection, despite studies in real-world settings showing a high safety profile in these patients. Additionally, to date, no clear evidence suggests that ICIs increase the risk of a SARS-CoV-2 infection [3]. Whether the administration of anti-PD1 drugs, by stimulating the immune system, could directly contribute to a more severe course of COVID-19, compared to that observed in patients not receiving immunotherapy, is currently an open question. The potential interplay between COVID-19 infection and treatment with immune-checkpoint inhibitors of patients with a melanoma is still unknown. Nevertheless, preliminary evidence and case reports suggest that anti-PD1 therapy does not worsen the course of COVID-19, allowing patients with cancer to continue their treatment [29]. In this Italian multicenter study by Pala et al., out of 169 patients with unresectable stage III or IV melanomas treated with immunotherapy, 104 continued without modifications, and among 15 patients showing symptoms compatible with COVID-19, only one tested positive on the nasopharyngeal swab [30].

## 6. Vaccination against COVID-19 in Patients with Cancer Receiving Active Therapy

Most currently authorized trials of vaccines against COVID-19 have not included patients with active malignancies. Hence, data on safety, tolerability, and efficacy in cancer populations are limited. However, these patients represent a high-priority subgroup for vaccination due to their higher risk of death from SARS-CoV-2 infection and, for this reason, vaccination is supported by the ESMO, the Society for Immunotherapy of Cancer (SITC), the Spanish Medical Oncology Society (SEOM), and the NCCN COVID-19 Vaccination Advisory Committee [31,32,33,34]. In Israel, a study was conducted on the efficacy of the BNT162b2 vaccine in 102 patients with solid tumors undergoing a systemic therapy. The most frequently used types of therapy were chemotherapy (29%), followed by immunotherapy (22%), and chemotherapy plus biological therapy (20%). Ninety percent of oncological patients tested positive for SARS-CoV-2 anti-spike IgG antibodies after the second vaccine dose. Furthermore, patients undergoing chemotherapy plus immunotherapy presented a median IgG titer that was significantly lower than the control group. Conversely, immune checkpoint inhibitors alone did not interfere with antibody production. To date, there is no evidence of the correlation between the efficacy/duration of protection and IgG titer; therefore, these serology data strongly support the current recommendations for the oncology population. Moreover, as these patients may not be able to develop a sufficient immune response themselves, it has become crucial that their caregivers are also vaccinated [35]. In an Italian center, 131 patients with cancer receiving active therapy (57% had skin cancers) and immunized against SARS-CoV-2 were compared to healthy individuals after two doses of mRNA-1273 (Moderna). The median values of anti-spike IgG were significantly higher for patients receiving immunotherapy compared to those receiving chemotherapy/targeted therapy [36]. Some researchers have hypothesized that the vaccine could hypothetically lead to an exaggerated immune response in immunotherapy recipients. Since vaccination may overload the immune system and trigger an important cytokine response, severe toxicity may damage the organs [24]. However, several studies evaluated that there were no new or exacerbated immune-related side effects in patients undergoing immunotherapy and receiving mRNA COVID-19 vaccines [37,38]. These data are also supported by the experience of the safety of influenza vaccination in the same population [39].

## 7. Lymph Adenopathy during Pandemic: Malignant Spread versus Benign Reaction to Vaccination

Unilateral axillary adenopathy is a potential side effect following COVID-19 vaccination (specifically the mRNA vaccines) and it was reported to appear 2 to 4 days after administration. During the clinical trials of Moderna, the average duration of lymphadenopathy was 1 to 2 days, whereas the Pfizer-BioNTech clinical trials showed an average duration of approximately 10 days [40,41]. This finding may be present in the staging or routine follow-up imaging of oncologic patients. Indeed, an FDG-avid lymphadenopathy, detected using a PET-CT scan, was described after COVID-19 vaccination and after an influenza vaccination [42,43]. Prieto et al. reported the case of a patient with a history of stage IIIA melanoma in the left deltoid region who underwent an image-guided biopsy of a right axillary lymphadenopathy that was discovered during the initial stage. A histological examination showed a reactive lymphoid tissue. Further investigations revealed that the patient had received the first dose of the Moderna vaccine in the right deltoid muscle 5 days prior to the PET-CT scan [44]. Sonographically detectable lymph node changes after COVID-19 vaccination have been described in patients with skin cancer attending tumor follow-up appointments. These lymphadenopathies resemble lymph node metastases in terms of enlargement, peripheral vascularization, and decreased echogenicity [45]. Given an ever-increasing worldwide vaccinated population, it will be important for oncologists and dermatologists to obtain a vaccination history to better interpret the results of imaging studies and to avoid invasive procedures that are not strictly necessary. Additionally, in tumor patients, the vaccination should be performed contralateral to the primary tumor to ensure the lowest possible chance of misdiagnosis. Ultimately, management should consider the probability of a malignant spread versus a benign reaction to recent vaccination. The factors to consider include the local site of the primary malignancy, common drainage pathways, time since vaccination (within or beyond 6 weeks after vaccination), prognosis, and overall risk profile [46].

## 8. Experiences in Our Dermatological Clinic

In our dermatological clinic in Turin, Italy, we retrospectively collected data on melanomas excised in May and June of the years 2017, 2018, 2019 and 2020 (Table 1).

This study aimed to highlight possible differences between the two-month period during the pandemic and the same timeframe in the previous three years, focusing on patient demographics and histological characteristics of melanomas. In the two months of 2020, there was an approximately 32% reduction in melanoma exeresis compared to the previous years. There were no gender-based differences but the average age was lower at 55 years—in the previous three years, the average age was greater than 60. The most frequently excised histotype in 2020 was superficial spreading melanoma (SSM), whereas in previous years it was melanoma in situ. The greatest Breslow index was recorded in 2020 with a thickness of 9 mm and, in the same year, the average thickness was 1.56 mm; although the average thickness was higher than the average in previous years, this difference was not statistically significant.

Concerning the sentinel lymph node (SLN) surgical interventions in our melanoma unit, we performed SLN biopsies in 41 patients in the first two months of the emergency; the average age was 55, four of whom were over 70. All patients received an antibiotic prophylaxis before the surgical procedure, the same treatment that was prescribed before the COVID-19 era. No COVID-19-related complications were observed, supporting the possibility of continuing surgical diagnostic procedures in patients with a melanoma if carefully managed and suspicious symptoms are strictly monitored [47].

At the beginning of the emergency period, 80 patients with a melanoma were receiving immune checkpoint inhibitors (ICIs) in our center (62 nivolumab and 18 pembrolizumab). Adjuvant therapy was administered to 31 subjects for disease-free stages III-IV, whereas 49 patients were treated for advanced metastatic disease. A total of 57 patients (71%) continued treatment without interruptions, whereas 16 postponed their therapy of one (14 patients; 17.5%) or two cycles (two patients; 2.5%). The remaining seven patients (9%) suspended treatment due to progression (*N* = 5), completion of schedule (*N* = 1) or were lost to follow-up (*N* = 1). Moreover, four patients started a new treatment during the pandemic. In our experience, at the time of writing, no patients under ICIs developed COVID-19 infection [48]. In the same period, there were 67 advanced metastatic patients with a melanoma receiving targeted therapy with BRAF and MEK inhibitors at our center: 58 patients were receiving dabrafenib and trametinib, six were receiving vemurafenib and cobimetinib, and three were receiving encorafenib and binimetinib. There were also 23 patients receiving adjuvant treatment for disease-free stage III exclusively with dabrafenib and trametinib. Despite the COVID-19 outbreak, we decided to maintain treatment in all patients due to the available clinical data on the increased relapse risk in subjects discontinuing target therapy. The symptoms of patients were strictly monitored to detect potential COVID-19 infection at an early stage, in addition to maintaining strict triage procedures at the hospital entrance. At the time of writing, no patients developed COVID-19 infection [49].

## 9. Conclusions

The pandemic significantly influenced dermatological practice and the management of skin tumors. Despite the growing importance of telemedicine, suspicious skin lesions require a face-to-face consultation, which can be completed safely by ensuring that all prevention measures for COVID-19 infection are followed.

Although a decrease in the early diagnoses of melanoma was observed, surgical interventions and systemic treatments for advanced cases were guaranteed, largely following the pre-pandemic-era international guidelines. Unfortunately, all cases with a diagnostic delay caused by the pandemic have a profound impact on healthcare procedures and costs, as well as having devastating consequences for patients.

Considering the high mortality rate of COVID-19 infection among patients with cancer and the safety of vaccines in those also undergoing systemic therapy, vaccination for these patients and their families is a priority.

Based on our experience, the choice to continue all diagnostic and therapeutic procedures in patients with a melanoma has proven to be safe, ensuring that the care these patients require continues during pandemics.

## Figures and Tables

**Table 1:** Demographic and histological features of melanomas excised in the two-month period from May to June in 2017, 2018, 2019, and 2020. MIS: melanoma in situ; SSM: superficial spreading melanoma; LMM: lentigo maligna melanoma; NM: nodular melanoma.

Year	N. of Melanomas Excised	Male	Female	Mean Age (Years)	Histotype	Breslow Thickness (Average)
2017	51	31	20	61	28 MIS 21 SSM 1 LMM 1 NM	1 mm
2018	41	20	21	62	25 MIS 11 SSM 4 LMM 1 NM	0.42 mm
2019	48	31	17	61	27 MIS 18 SSM 2 LMM 1 NM	0.99 mm
2020	32	16	16	55	13 SSM 12 MIS 3 LMM 2 NM 1 nevoid melanoma 1 acral melanoma	1.56 mm
